# Cellular responses in rainbow trout *Oncorhynchus mykiss* to experimental *Anisakis simplex* infection

**DOI:** 10.1007/s00436-025-08565-2

**Published:** 2025-09-25

**Authors:** Kaan Kumas, Bahram Sayyaf Dezfuli, Emanuela Franchella, Yajiao Duan, Per Walter Kania, Kurt Buchmann

**Affiliations:** 1 Faculty of Health and Medical Sciences, Department of Veterinary and Animal Sciences, University of Copenhagen, Frederiksberg, Denmark; 2 Department of Life Sciences and Biotechnology, University of Ferrara, Ferrara, Italy

**Keywords:** Fish, Nematodes, Immunity, Protection

## Abstract

Fish in wild and cultured populations may be infected with numerous types of pathogens but the host responses vary dependent on both host and parasite species. The present study demonstrates how an experimental infection with endoparasitic nematode larvae (*Anisakis simplex*) induces cellular and humoral immune responses in rainbow trout *Oncorhynchus mykiss*. The nematode larvae invaded the peritoneal cavity of the fish following oral administration and became encapsulated by a range of host cells including macrophages, neutrophils, mast cells, fibroblasts, and lymphocytes. The main part (92.7%) of the recovered larvae was located in the body cavity and 51.3% along the pyloric caeca with only few in or on the stomach, liver, spleen, swim bladder, and musculature. The cellular reaction was documented by transmission electron microscopy (TEM) and histochemistry. Real time quantitative PCR (qPCR) showed that a series of immune-relevant genes in the host spleen became regulated by the infection. Thus, *A. simplex* induced downregulation of immune-genes (encoding IgD and lysozyme) and upregulation of the gene encoding the immune-regulating cytokine IL-10. Nematode molecules influencing the antiparasitic host reactions are discussed.

**Supplementary Information:**

The online version contains supplementary material available at 10.1007/s00436-025-08565-2.

## Introduction

Teleost fishes in natural and farmed populations may be infected with a wide range of pathogens (Buchmann and Bresciani 1997; Setyawan et al. 2019; 2020; Falkenberg et al. 2024), and these viral, bacterial, or parasitic pathogens will initiate a host response as documented by numerous studies (Utke et al. 2008; Olsen et al. 2011; Kumari et al. 2015; Dickerson and Findly 2017; Castellano et al. 2020). However, the main part of controlled investigations using helminths as pathogens in various vertebrate hosts indicates that nematodes modify the host response to increase their survival (Maizels et al. 2018). Infections with anisakid endoparasites, such as *Anisakis simplex* and *Contracaecum osculatum* nematode larvae, are believed to modulate a range of immune genes in the fish host (Larsen et al. 2002; Bahlool et al. 2013; Marnis et al. 2020). Similarly, European eel *Anguilla anguilla* are often heavily infected by *C. rudolphii*, and although cellular responses are noted, the parasite seems to survive well (Sayyaf Dezfuli et al. 2024; 2025). Nematode larvae of the species *A. simplex* are prevalent in wild fish stocks (Mattiucci et al. 2018; Cipriani et al. 2024; EFSA 2024), including clupeids (Levsen and Lunestad 2010) and salmonids (Murphy et al. 2010; Mo et al. 2021). The life cycle in marine waters comprises marine mammals as the definitive hosts and small crustaceans, squids, and fish as paratenic hosts carrying the infective third stage larva, which after reaching the mammals’ gastro-intestinal tract molts twice and attains the adult reproductive stage (Mattiucci et al. 2018). In the wild fish, the larvae are often found encapsulated by host cells (Sayyaf Dezfuli et al. 2021a) but the nature of the encapsulation process in rainbow trout needs further exploration under controlled conditions. Experimental infection models, which may be applied for the purpose, have been established using salmonids as hosts and *A. simplex* as pathogen (Tojo et al. 1994; Larsen et al. 2002; Bahlool et al. 2013; Kumas et al. 2025). The present study aims to elucidate how a primary experimental infection of rainbow trout *O. mykiss* with *A. simplex* larvae elicits a cellular response and which immune genes the fish applies in the process. Fish were infected by *A. simplex* third stage larvae (L3) by oral administration. Controls were sham-infected. During the process, we investigated cellular immune response mechanisms by TEM and histopathological and histochemical methods, and we elucidated a possible involvement of immune factors by performing real-time quantitative PCR (qPCR) on spleen samples of the fish.

## Materials and methods

### Fish

An outbred population of rainbow trout (*Oncorhynchus mykiss)* was produced by the company Aquasearch ova ApS (Jutland, Denmark) and transported (as eyed eggs) to the hatchery. They were incubated for hatching at 7–8 ℃, and after hatching, yolk sac larvae reached the swim up stage 14–21 days later and were then fed with commercial fry feed (Biomar, Denmark), transferred to 1-m^3^ tanks, and reared at 12–13 ℃ in a pathogen-free recirculated aquaculture system (AquaBaltic, Bornholm, Denmark). Fish were then transferred to the experimental fish infection facility at the University of Copenhagen, Section of Aquatic Pathobiology and Parasitology, Frederiksberg Campus, and kept in 200-l tanks containing municipal tap water (CaCO_3_ > 450 mg/L) at 16 °C. Water was recirculated by internal bio-filters (Eheim, Germany) and aerated (100% oxygen saturation). NH_3_, NO_2_, and NO_3_ levels were monitored daily by water quality strips (Tetra, Germany). The fish were acclimatized for 2 months in the facility before experimentation. Commercial trout feed (Inicio, Biomar, Denmark) was used for feeding the fish (2% biomass per day). The mean body length of the trout was 9.7 cm (SD 0.7) and the mean body weight was 10.4 g (SD 2.4) at the start of the experiment.

### Recovery of parasites for experimental infection of rainbow trout

#### Nematode larvae

**Fig. 1:**
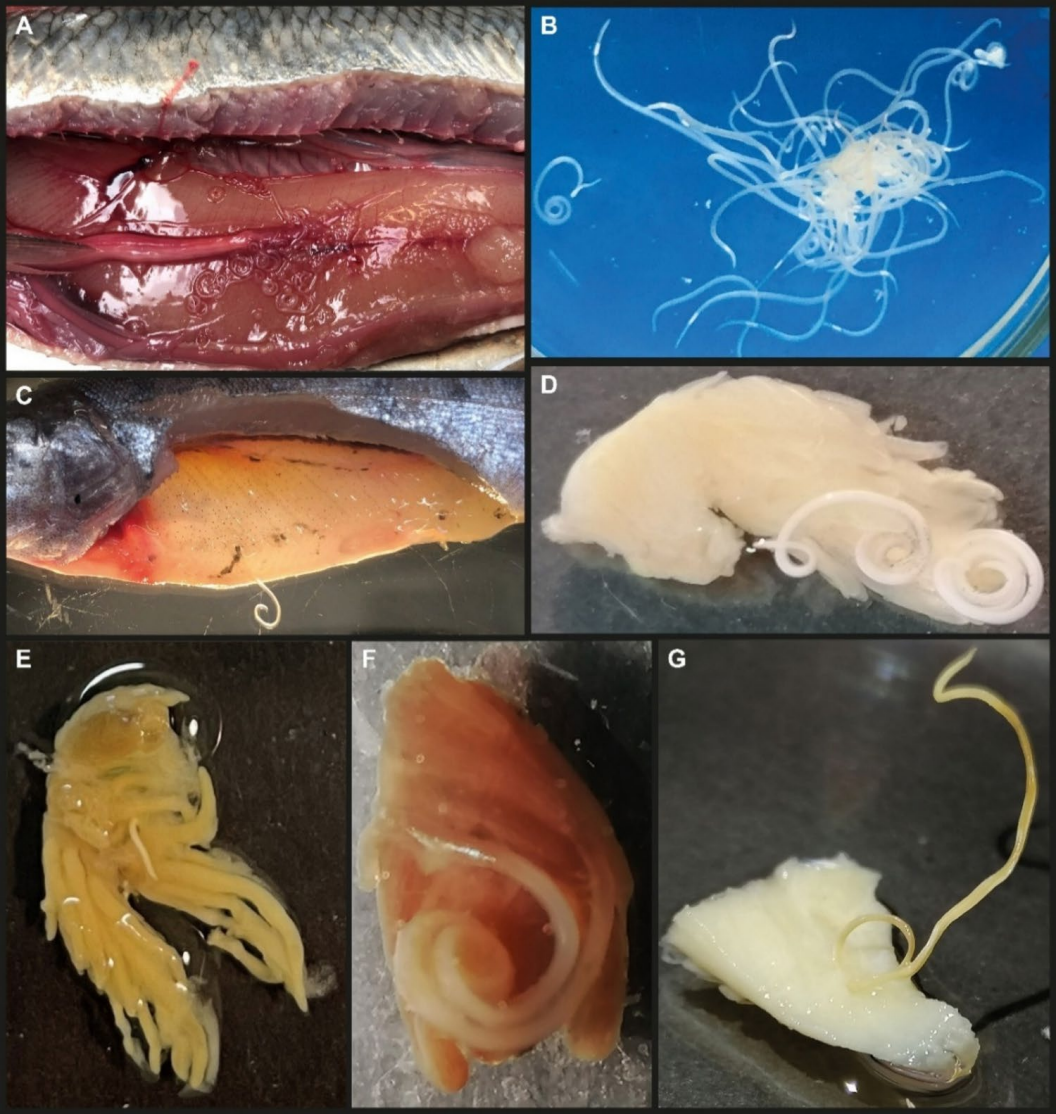
**A** *Anisakis simplex* 3rd stage larvae in the body cavity of Atlantic herring *Clupea harengus*, serving as source of the experimental infection. **B** Live *A. simplex* larvae in Petri dish recovered from herring to be used for experimental infection of rainbow trout. **C** Experimentally infected rainbow trout with larva penetrating the right-side ventral musculature. **D** Section of intestine with larva attached to external surface. **E** Pyloric caeca with penetrating larva. **F** Rainbow trout liver with partly penetrating larva. **G** Section of right-side musculature with attached larva

Third stage larvae of *Anisakis simplex* were recovered from Atlantic herring *Clupea harengus*, which were captured by a commercial trawler in the North Sea (ICES area A4). Fish were dissected and opened by ventral and lateral incisions exposing the body cavity (Kumas et al. 2024a) (Fig. 1A). Nematodes were isolated from the body cavity using soft forceps and placed into tap water at room temperature for a few hours, which allowed the larvae to escape from the host encapsulation (Fig. 1B). Only actively moving parasites were recovered. A subsample of the isolated nematode larvae was preserved in 96% ethanol for the morphological and molecular identification of the species.

### Experimental design

A total of 30 rainbow trout were used in the experimental study. The immune expression study included 20 fish (2 × 5 fish exposed to infection (two worms per fish) and 2 × 5 fish kept as non-infected controls). The histological study used 10 fish (2 × 5 rainbow trout exposed to ten worms per fish). The latter infection allowed us to collect sufficient material (larvae encapsulated in host organs and tissues) for TEM and histochemical/histopathological studies.

### Infection procedure

For the nematode infection procedure, we selected nematode larvae exhibiting viability and motility (Fig. 1B) according to Kumas et al. (2024b), and care was taken to avoid any worm damage during the infection. *Anisakis* challenges were carried out according to Larsen et al. (2002) and Kumas et al. (2025) by placing, with the aid of forceps, nematode larvae in the stomach of anaesthetized fish (MS222, 40 mg/L). A group of ten fish (2 × 5) was kept non-infected and non-exposed throughout the experimental period (control group). Another group of ten fish (2 × 5) was used for the immune gene expression investigation. Each of these was infected with two nematode larvae. A third group (2 × 5 fish) was used for the histological (light microscopy and transmission electron microscopy) investigation. They were each infected with ten nematode larvae.

### Dissection and sampling

Two weeks after the challenge, the experiment was terminated by euthanizing all the fish by immersion into a solution of MS222 (300 mg/L). The rainbow trout used were opened by longitudinal cuts (ventrally and laterally), and the belly flap was removed, which exposed the body cavity and internal organs.

#### Nematode recovery

**Fig. 2:**
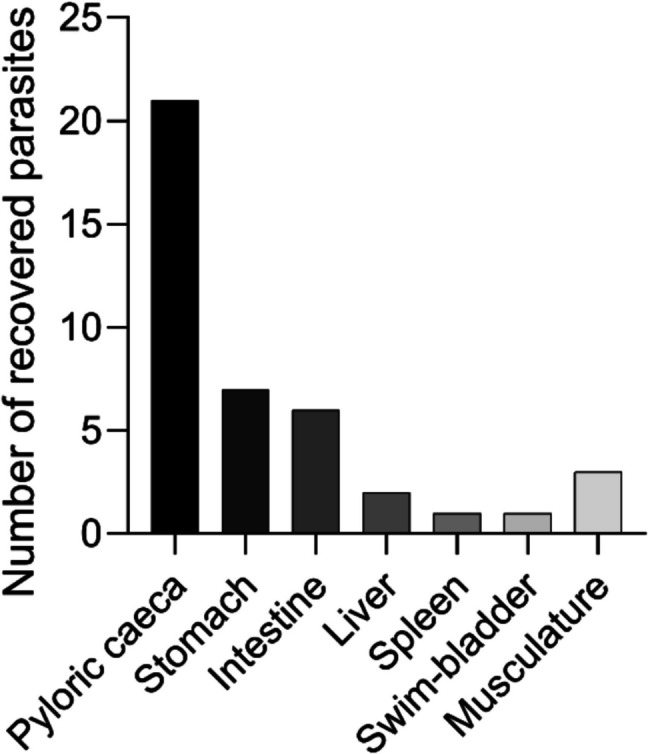
Diagram depicting the number of *Anisakis simplex* 3rd stage larvae recovered from the different internal organ locations in experimentally infected rainbow trout. Based on 10 rainbow trout each experimentally infected with 10 parasites. Out of 100 larvae used for infection a total of 41 were recovered from the infected rainbow trout

The organs in the body cavity (liver, spleen, stomach, pyloric caeca, intestine) of the ten rainbow trout (to be used for histopathological investigation), each of which were exposed to ten nematode larvae, were inspected for the presence of *A. simplex* larvae (Fig. 1C to G). The position and number of worms were recorded for each infected organ (Fig. 2). Organs and tissues with *Anisakis* parasites were taken for histopathological and histochemical analysis (aiming at detecting and characterizing cellular reactions to the *Anisakis* worms). Thus, infected organs were placed in neutral 10% formaldehyde for 24 h and transferred to 70% ethanol (see below). Additional ten nematode larvae encapsulated in the body cavity along the pyloric caeca were fixed in glutaraldehyde for TEM studies (see below). Following removal of macroscopically visible worms, the remaining parts of the fish were exposed to pepsin digestion (Buchmann 2007) in order to reveal any hidden worms in organs and musculature.

#### Immune organ sampling for qPCR

Aiming at describing the expression of immune genes in the fish, we sampled spleens from the ten fish, which had been exposed to two nematode larvae each. The organ was dissected aseptically and preserved in RNAlater. The material was later subjected to RNA purification, cDNA production allowing subsequent real-time quantitative (qPCR) gene expression analyses.

#### Organ sampling for histology

Pieces of organs (approx. 15 × 15 × 10 mm) containing *A. simplex* larvae as well as 6 pieces of each uninfected *O. mykiss* organs were excised, photographed, and then fixed in 10% neutral buffered formalin for 24 h.

#### Organs for TEM

For transmission electron microscopy (TEM), we recovered subsamples of pyloric caeca and associated tissues with nematode larvae from parasitized rainbow trout. Corresponding organs from ten non-infected control trout were taken. In brief, pieces of 7 × 7 mm in size were fixed in cold 2.5% glutaraldehyde in 0.1 M sodium cacodylate buffer for 3 h as described in Sayyaf Dezfuli et al. (2024).

#### Histopathology and histochemistry

Organs fixed in formalin were transferred to 70% ethanol after 24 h. Each organ sample (infected or non-infected trout) was dehydrated, embedded in paraffin wax, and cut with routine histological techniques. Multiple 5-µm sections were taken from each tissue block, stained with either Alcian Blue-Haematoxylin and Eosin (AB/HE), Alcian Blue 8 GX pH 2.5 and Periodic Acid Schiff (AB/PAS) or Giemsa, examined, and photographed using a Nikon Microscope ECLIPSE 80i (Nikon, Tokyo, Japan).

#### Transmission electron microscopy TEM

The glutaraldehyde fixed tissues were post-fixed in 1% osmium tetroxide for 2 h and then rinsed and stored in 0.1 M sodium cacodylate buffer containing 6% sucrose for 12 h. Thereafter, the tissue pieces were dehydrated through a graded acetone series and embedded in epoxy resin (Durcupan ACM, Fluka, Buchs, Switzerland). Semi-thin sections (1.5 µm) were cut on a Reichert Om U 2 ultramicrotome (Reichert, Vienna, Austria) and stained with toluidine blue. Ultra-thin sections (90 nm) were stained with 4% uranyl acetate solution in 50% ethanol and Reynold’s lead citrate and then examined using a Talos L120C transmission electron microscope (Thermo Fischer Scientific, MA, USA).

#### Morphological and molecular identification of parasites

A subsample of isolated parasites, preserved in 96% ethanol at 4 °C, was divided into three (anterior, middle, caudal) pieces. The anterior and caudal parts were used for morphological identification and the middle part was used for molecular identification. Parasite parts were mounted on microscope slides with mounting medium Aquatex® (Merck, Germany) (Supplementary file S1). Molecular identification of parasites was performed by targeting rDNA (both for trematodes and nematodes) and mtDNA (only for nematodes) by PCR and subsequent sequencing. QIAamp DNA Mini Kit (Qiagen, Denmark) was used to purify genomic DNA according to the manufacturer’s instructions. PCR was performed in 60 µl volumes composed of 28.6 µl RNase-free water (Fisher Scientific, Denmark), 6 µl dNTP mix (Applied Biosystems™ GeneAmp™ dNTP Blend (100 mM), Thermo Fisher Scientific, Denmark), 0.6 µl DNA polymerase, 6 µl 10 × reaction buffer, 1.8 µl 50 mM MgCl_2_ (BIOTAQ DNA Polymerase, Saveen & Werner ApS, Denmark), and 6 µl of forward and reverse primers (both 10 mM) (Tag Copenhagen, Denmark) (Table 1).

**Table 1:** Primers and PCR conditions. In general, the PCR conditions consisted of pre-denaturation at 95 °C for 5 min, 45 amplification cycles of denaturation at 95 °C for 30 s/annealing at primer specific temperature for 30 s/elongating at 72 °C for primer-specific time followed by a post-elongation step at 72 °C for 7 min. The PCR for the mitochondrial gene *cox2* encoding for cytochrome c oxidase subunit 2 used a touchdown procedure by gradually lowering the annealing temperature (2 cycles at 57 °C, 2 cycles at 55 °C, 2 cycles at 54 °C, 3 cycles at 53 °C, 3 cycles at 52 °C, 3 cycles at 51 °C, and 35 cycles at 50 °C). The BD1 and NC2 primers were situated at the end of 18S rRNA and the start of 28S rRNA encoding regions, respectively. Thus, they produced an amplicon consisting of 18S rRNA (partly)-ITS1-5.8S rRNA-ITS2-28S rRNA (partly) denoted ITS-region; ITS indicates internal transcribed spacer

Primer	Sequence 5′ end to 3′ end	Target	Annealing temperature	Amplicon size	Elongation time	Reference
211F	ttttctaagttatatagattgrtttyat	*cox2*	Touchdown from 53 °C to 46 °C	970 bp	30 s	Nadler and Hudspeth (2000)
210R	caccaactcttaaaattatc					
BD1	gtcgtaaca aggtttccgta	ITS-region	54 °C	630 bp	60 s	Galazzo et al. (2002)
NC2	ttagtttcttttcctccgct					Gasser et al. (1993)

The PCR products were visualized by 1.5% agarose gel-electrophoresis. Products were purified using the Illustra™ GFX™ PCR DNA and Gel Band Purification Kit (VWR International A/S, Denmark). The mitochondrial gene *cox*2 encoding the cytochrome c oxidase subunit 2 and the ribosomal DNA region ITS were amplified using the primers and PCR conditions specified in Table 1. PCR products were sequenced at Macrogen Europe, Netherland, and analyzed using the software CLC Main Workbench v20.0.4 (Qiagen, Denmark). Sequences were submitted to GenBank (NCBI).

#### Immune gene expression

Spleens were sampled and fixed in RNAlater (Merck, Denmark), placed at 4 °C for 24 h and transferred to −20 °C until further processing. Spleens were homogenized with 2-mercaptoethanol in TissueLyser II at 20 Hz for 2 min using stainless steel beads, 5 mm (200) (Qiagen, Denmark). RNA purification was performed by the use of GenElute™ Mammalian RNA Kit (Merck, Denmark). DNase (AMPD1, Merck, Denmark) treatment was applied to all samples and 50 µl purified RNA was mixed with 5 µl 10 × reaction buffer and DNaseI and incubated at 37 °C for 1 h, whereafter a 5 µl stop reaction (EDTA) was added and incubated 10 min at 65 °C. Then, products were visualized by 1.5% agarose gel-electrophoresis and the RNA quality and quantity was evaluated using a NanoDrop 2000 spectrophotometer (Saveen & Werner ApS, Denmark). cDNA syntheses were performed using the kit TaqMan™ Reverse Transcription Reagents (Thermo Fisher Scientific, Denmark) in 20 µl reaction volumes and using 1000 ng of RNA. Samples without transcriptase were included as negative controls and samples with only reagents and RNase-free water as positive control. PCR conditions were as follows: one cycle of annealing at 25 °C for 10 min, elongating at 37 °C for 60 min, and inactivation at 95 °C for 5 min. Eighty microliters of RNase-free water was added to the samples after the cDNA synthesis. Expression of genes encoding the following host molecules was described: β-actin, Arp, Saa, Elf α, C3, Cathelicidin 1, Cathelicidin 2, IFNɤ, IgDm, IgDs, IgM, IgT, IL-1B, IL-2, IL4/13a, IL-6, IL-8, IL-10, IL-12, IL-17A/F2, IL17/C1, IL17/C2, IL-22, Lysozyme, TCRβ, TGFβ, and TNFα genes are tested. These molecules represent broadly innate and adaptive immune reactions in rainbow trout (Kania and Buchmann 2024; Kumas et al. 2025). Primers and probes are listed in Suppl. File S2. References listed with each primer describe and explain the production and validation of each primer and probe set. Each qPCR reaction was composed of 2.5 µl cDNA, 6.25 µl Brilliant III Ultra-Fast QPCR Master Mix (AH Diagnostics AS, Denmark), 1.0 µL primer–probe mixture (10 µM forward primer, 10 µM reverse primer), TaqMan probe (5 µM), and 2.75 µl RNase-free water. Gene expressions were carried by one cycle pre-denaturation at 95 °C for 10 min and 40 cycles of denaturation at 94 °C for 10 s with a combined annealing/elongation process at 60 °C for 15 s. The software NormFinder (Andersen et al. 2004) was used to find the best combinations of reference genes, and the genes encoding β-actin, acidic ribosomal phosphoprotein P0 (ARP), and elongation factor (ELF)1-α were then used as reference genes.

### Statistics and calculations

Infection parameters as abundance, mean intensity, and prevalence values calculated according to Bush et al. (1997). As all qPCR assays used have efficiencies within 100 ± 5% (Schmittgen and Livak 2008), the relative fold changes were calculated using the simplified 2^−ΔΔCq^ method (Livak and Schmittgen 2001). The results were analyzed using the ΔCq (= -log^2^ transformation of the fold) in two-way ANOVA. Significance was considered when both *p* < 0.05 and fold change of at least 2. Levels (2^−ΔCq^) were used to depict the results. Due to their exponential nature, the geometrical means and geometrical standard error means (GSE) of the levels were used. GraphPad Prism 10.2.3 (USA) was used for statistical analysis.

## Results

### Parasite identification

The nematode larvae recovered from the herring and used for infection of trout were all identified by morphology to genus level (characterized by larval boring tooth, excretory pore anteriorly to nerve ring, lack of ventricular appendage and intestinal caecum) (Supplementary File S1) and molecular methods (species level) as *A. simplex *sensu stricto (GenBank accession numbers were achieved and included for ITS nos. PV828134-PV828144 and for *cox2* nos. PV862037 → PV862046).

### Infection levels (prevalence, mean intensity, and abundance)

Prevalences were 100% for nematode larvae in fish exposed to ten larvae each. The mean intensity was 4.1 parasites per fish (abundance 4.1). In fish each exposed to two larvae, the prevalence was 80% and the mean intensity 1.5 (abundance 1.2).

### Location of *A. simplex* larvae in host fish

The main part of the recovered nematode larvae (38 out of 41 corresponding to 92.7%) was located in the body cavity along mesenteries and organs of rainbow trout, where they were found encapsulated in cellular material of the host. The pyloric caecum region harboured 51.3% (21 out of 41 larvae), the intestine region 14.6% (6 of 41), the stomach wall 17.1% (7 of 41), the liver 4.9% (2 of 41), the spleen 2.4% (1 of 41), the swim bladder 2.4% (1 of 41), and the musculature 7.3% (3 of 41) (Figs. 1 and 2). The nematode larvae were detected quite efficiently by visual inspection under the dissection microscope and only 2 out of 41 recovered worms were found after pepsin/HCl digestion of the individual organs (1 in pyloric caeca, 1 in musculature), which corresponds to 95% recovery rate by visual inspection.

### Histopathology

**Fig. 3:**
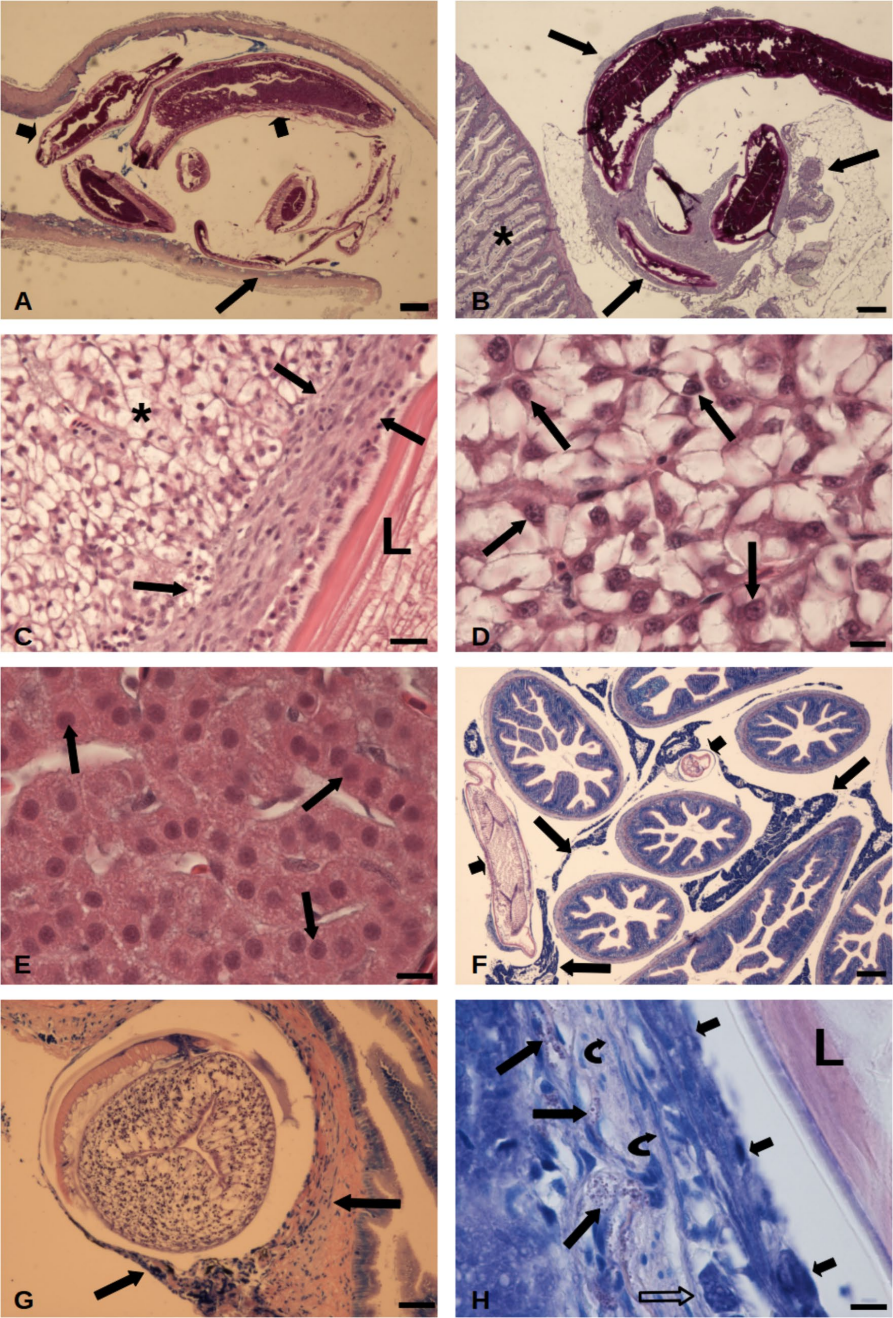
Histological sections of infected/uninfected visceral organs of *Oncorhynchus mykiss.* **A** Intestine of *O. mykiss* infected with two specimens of *Anisakis simplex* larvae (short arrow), lack of intestinal folds (long arrow) is evident, stain: Alcian Blue-PAS (ABPAS); scale bar: 200 µm. **B** *A. simplex* larva encysted on outer surface of the intestine (asterisk), apical part of the worm is embedded in pancreatic tissue (arrows), stain: AB/PAS; scale bar: 200 µm. **C** Between nematode larva (L) and liver (asterisk) presence of multilayer capsule (arrows) is visible, note rarefaction of the hepatocytes, stain: haematoxylin and eosin (H&E), scale bar: 25 µm. **D** High magnification of infected liver of rainbow trout, eccentric position of the nuclei (arrows), and rarefaction of the cytoplasm are evident, Stain: (H&E), scale bar: 10 µm. **E** Normal hepatic tissue from control group, central position of the nuclei (arrows), and no rarefaction of the cytoplasm can be seen, Stain: (H&E), scale bar: 10 µm. **F** Sections of *A. simplex* larvae (short arrows) between pyloric caeca, parasite body in contact with exocrine acinar pancreatic tissue (long arrows), stain: Giemsa, scale bar: 100 µm. **G** Image shows the cephalic part of *A. simplex* larva encysted in submucosal layer of a caecum, note the presence of capsule (arrows) around the larva, Stain: Giemsa, scale bar: 50 µm. **H** High magnification of interface region between larva (L) in the caecum submucosal layer, epithelioid cells (short arrows) of inner layer, in middle layer the fibroblasts (curved arrows) and macrophages (empty arrow) immersed in a mesh of collagen, in outer layer mast cells (long arrows) are visible, Stain: Giemsa, scale bar: 10 µm

The nematode larvae occupied different microhabitats (Fig. 1C to G). Two *A. simplex* larvae were located within the intestine of rainbow trout. A portion of one worm was located on top of the intestinal folds with resulting erosion and damage of their integrity (Fig. 3A and B). No host immune cells were recognized within the intestinal mucosal layer. In very few trout, *A. simplex* larvae were attached on the outer surface of the anterior intestine and a portion of parasite was embedded in the pancreas (Fig. 3B). In a limited number of infected trout, *A. simplex* was found in the liver (Fig. 3C). A three-layer capsule separated the larval body from the organ parenchyma. The innermost layer of the capsule was formed by fibroblasts and the middle layer contained fibroblasts immersed in a mesh of collagen fibres and a very few mast cells, whereas the outer layer was formed by collagen fibres and some macrophages. In the infected liver, the organ lost its normal architecture, and hepatocyte was recorded as with eccentric nucleus, cytoplasmic rarefaction, and cell swelling (Fig. 3C and D). The livers of control fish showed no abnormal structures and the hepatocytes had spherical central nuclei with no rarefaction of the cytoplasm (Fig. 3E). The vast majority of *A. simplex* larvae encapsulated along the pyloric caeca was in contact with or surrounded by exocrine acinar pancreatic tissue (Fig. 3F). In some histological sections, the cephalic part of the nematode was inside the caeca, encysted in the submucosal layer and encircled by a capsule (Fig. 3G). Detailed observations of this three-layered capsule revealed an inner layer of epithelioid cells in close proximity to the parasite cuticle. It was followed by a middle layer containing fibroblasts scattered among a mesh of collagen fibres and some macrophages. The outer layer was composed of some mast cells, collagen fibres, and a few macrophages (Fig. 3H). Sometimes in the middle and outer layer, the occurrence of neutrophils and lymphocytes was observed (see below).

### TEM

**Fig. 4:**
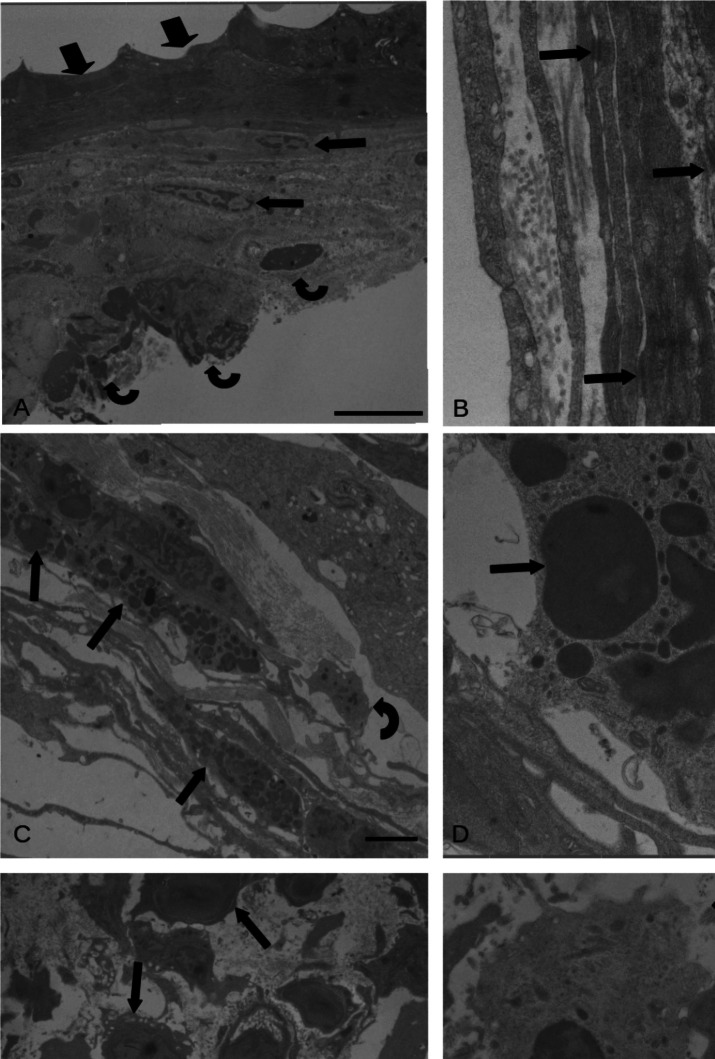
Transmission electron micrograph of *Oncorhynchus mykiss* pyloric caeca infected with larvae of *Anisakis simplex*. **A** Interface region between *O. mykiss* caecum and *A. simplex* larva; the inner layer consists of epithelioid cells (arrow heads) with electron-opaque cytoplasms, middle layer with fibroblasts with elongated nuclei (arrows) immersed in mesh of collagen, and outer layer with macrophages (curved arrows); scale bar: 5 µm. **B** High magnification of the inner layer, occurrence of desmosomes (arrows), and fragment of rough endoplasmic reticulum (curved arrows) are evident; scale bar: 0.5 µm. **C** Middle layer of the capsule, some macrophages (arrows) scattered among fibrotic tissue and a mast cell (curved arrow) in contact with collagen; scale bar: 2 µm. **D** High magnification of a macrophage, note electron-dense undefinable material inside the vesicles in cytoplasm (arrows); scale bar: 1 µm. **E** Occurrence of lymphocytes (arrows) and neutrophils (curved arrows) in the middle layer is visible; scale bar: 5 µm. **F** High magnification of a neutrophil (curved arrow) with heterochromatic nucleus and small elongated granules with different distribution of fibrils in cytoplasm. A lymphocyte (arrow) with heterochromatin nucleus and electron-dense cytoplasm, well-developed rough endoplasmic reticulum (empty arrow), and many dark elongated mitochondria (left side of nucleus); scale bar: 1 µm

Transmission electron microscopy observations were carried out only on infected pyloric caeca because this organ appeared to be the preferred site for migration of the vast majority (51.3%) of the larvae after infection. A capsule formed by host immune cells was recognized around the *A. simplex* larva encysted on the surface of pyloric caecum. The inner-most zone of the capsule was in close proximity of nematode cuticle and was formed by an epithelioid cell layer. These epithelioid cells were flattened, with very electron-opaque cytoplasm (Fig. 4A) and desmosomes between adjacent cells (Fig. 4B). The epithelioid cells resembled macrophages with large cytoplasmic and nuclear volume, with few mitochondria and well-developed rough endoplasmic reticulum (RER) and interdigitations. In these cells, cytoplasmic borders were indistinct (Fig. 4B). Presence of necrotic epithelioid cells was common. The middle layer of the capsule was formed by fibroblasts (Fig. 4A) scattered among a rich mesh of collagen, with spread mast cell and macrophage occurrence (respectively Fig. 4C and D). Indeed, within the middle layer, neutrophils and lymphocytes were noticed (Fig. 4E). Neutrophils had round, oval-shaped heterochromatic nuclei. The cytoplasm with many small, elongated granules with different distribution of fibrils (Fig. 4F) as well as small round mitochondria, and free ribosomes scattered in cytoplasm. Lymphocytes had electron-dense cytoplasm, big round shape heterochromatic nucleus and well-developed RER, and few oval mitochondria (Fig. 4F). The outer layer of the capsule around the *A. simplex* larva was formed by very large macrophages encircled with fibrotic tissue (Fig. 4C). Frequently, the macrophages in the capsule contained electron-dense material, some swollen membranes of the RER, and no other distinguishable cytoplasmic organelles (Fig. 4D).

### Immune gene expression

**Fig. 5:**
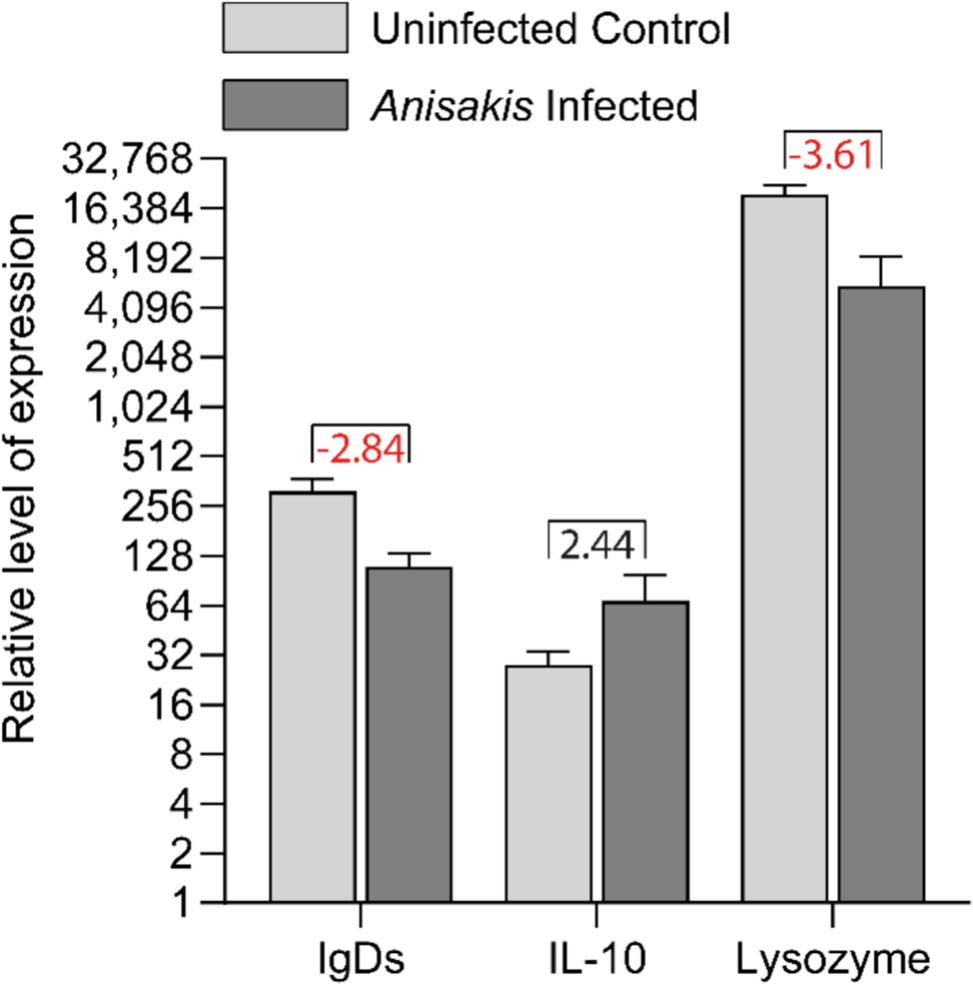
Immune gene expression levels in spleens of rainbow trout infected with *Anisakis simplex.* Results for three significantly regulated genes are shown. Columns show geometrical means (levels of gene expression). Brackets indicate significant difference. The numbers inside indicate the relative fold change. Levels were calculated as 2^−ΔCq^. The error bars indicate the geometrical standard error of mean

The rainbow trout exposed to *Anisakis* larvae showed significant regulation of three immune genes (*igds*,* lysozyme*,* il-10*) (Fig. 5). A comprehensive overview of all genes analyzed can be found in the Supplementary File S3. Comparing *Anisakis* infected to *Anisakis* non-infected fish showed downregulation of the genes *igds* and *lysozyme* and upregulation of the immune-regulating cytokine gene *il-10* (Fig. 5, Supplementary File S3).

## Discussion

The present study has elucidated the cellular responses elicited in rainbow trout following an experimental infection with the endoparasitic nematode larva *A. simplex.* This parasite is known to infect a wide range of mainly marine fish and elicit cellular responses (Sayyaf Dezfuli et al. 2021a, b). However, when performing an experimental challenge of previously non-infected fish and at the same time performing an analysis of immune gene expression, we have aimed at providing a deeper insight into the cellular immune responses to nematode larvae. The immune gene expression and regulation was minor in fish exposed to this nematode and far weaker compared to immune gene expression in trout exposed to viral pathogens (Utke et al. (2008), bacterial pathogens (Raida and Buchmann 2007; Deshmukh et al. 2013; Kumari et al. 2015; Castellano et al. 2020; Kania and Buchmann 2024), or protozoans (Olsen et al. 2011). Downregulation of the genes encoding an adaptive immune factor (the antibody IgD) and an innate defence molecule (the bactericidal lysozyme) and simultaneous upregulation of the gene *il-10* encoding the immune-regulating (immune-suppressing) cytokine IL-10 suggests that the nematode larva may modulate host immunity to increase survival after infection. This would align with observations in a wide range of other vertebrates infected with helminths (Maizels et al. 2018). Despite the nematode invasion of central organs in rainbow trout, the immune gene regulation was surprisingly limited and mainly downregulated. Following bacterial infection, rainbow trout will often exhibit a classical and marked inflammatory reaction and upregulate a wide range of immune molecules (interleukins, acute phase reactants, complements factors, and subsequently immunoglobulins) (Kania and Buchmann 2024). The array of immune genes tested, comprising signal molecules and effector molecules, in this work is able to map wide-spread interactions between innate and adaptive responses generally activated upon a bacterial infection (Deshmukh et al. 2013). Therefore, it is surprising that a penetration process of the relatively large nematode larva (20–25 mm in length) into the body cavity of rainbow trout was associated with downregulation of genes *igds* and* lysozyme.* On the other hand, we showed that rainbow trout host cells encapsulated the nematode larvae with active involvement of epithelioid cells, lymphocytes, neutrophils, mast cells, and macrophages. The cellular action seems to be local as previously suggested by Marnis et al. (2020) studying the nematode larva *Contracaecum osculatum* in liver of cod *Gadus morhua*. It may therefore be speculated that the cells encapsulating the larva are responding to immune regulating factors released by the parasite (Kumas et al. 2025). Nematode parasites of the fish damage host organs in different ways, including tissue eruption caused by mechanical injury (Molnár et al. 2019; Sayyaf Dezfuli et al. 2021b). Anisakid nematodes use several fish species as their paratenic hosts and commonly infect the liver, skeletal muscle, and gonad (Mattiucci et al. 2018; Sayyaf Dezfuli et al. 2021a; 2025; Cipriani et al. 2024). In addition, this study showed that the vast majority of *A. simplex* larvae were recovered from the surface of pyloric caeca. The direct impact on organs and tissues was thereby limited but still the host encapsulated the parasite. Fish react to extraintestinal helminths by forming connective encapsulation, which are focal chronic inflammatory lesions (Buchmann 2012; Buchmann and Mehrdana 2016; Behrens et al. 2023; Sayyaf Dezfuli et al. 2024). Worm encapsulation is a reciprocal adaption between the parasite and host immune response (Buchmann 2012; Sayyaf Dezfuli et al. 2024), and anisakid larvae encysting on fish organs are able to survive for extended periods locally if they downregulate the synthesis of immune molecules responsible for parasite expulsion (Marnis et al. 2020). With reference to fish visceral organs, often they are able to encircle the pathogen/parasite with a capsule formed by different types of host immune cells (Sayyaf Dezfuli et al. 2021b). Immune cells encountered in pyloric caeca around *A. simplex* larvae were metabolically active cells, such as macrophages, and less active cells, such as epithelioid cells (Secombes and Chappell 1996; Sayyaf Dezfuli et al. 2024). Epithelioid cells are morphologically similar to epithelial cells; they are transformed macrophages that form upon persistent inflammatory stimulation (Secombes and Chappell 1996; Gauthier et al. 2004; Ferguson 2006). Herein, necrotic epithelioid cells close to *A. simplex* larvae were also noticed; the occurrence of necrotic epithelioid cells in the inner layer of granulomas in close proximity to other nematode larvae has been widely reported (Molnàr 1995; Sayyaf Dezfuli et al. 2021a, b; 2024). Another type of cells occurred in the capsule around *A. simplex* larvae were fibroblasts; it seemed that they impede the penetration of parasites into host organs (Buchmann 2012), and under pathological conditions, fibroblasts contribute to the healing of damaged tissues (Secombes and Chappell 1996; Schuster et al. 2023). Fibroblasts in the capsules around other nematode species were previously recognized (Molnár et al. 2019; Behrens et al. 2023; Sayyaf Dezfuli et al. 2024). In addition, lymphocytes were observed within the capsule around the *A. simplex* larvae, a common finding around tissue nematodes (Molnar 1995; Molnár et al. 2019; Behrens et al. 2023). Another type of cell encountered in the present study is neutrophils, which are highly motile cells crucial for acute inflammatory responses and serve as the first line of defence against pathogens (Harvie and Huttenlocher 2015; Havixbeck et al. 2016; Buchmann 2022). Macrophages were prevalent around *A. simplex* and they may link adaptive and innate immunity. Some forms are part of the innate early defence exerting phagocytosis of intruders and some other forms serve as antigen presenting cells in the initiation of adaptive responses. A certain form contains melanin, is observed as single macrophages or may be organized in groups, and is then named as melano-macrophage aggregates (MAs) (Agius and Roberts 2003; Stosik et al. 2019). This cell type has been found associated with adverse conditions in fish such as starvation, chemical exposure, aging, and infectious diseases (Couillard et al. 1999; Agius and Roberts 2003; Ying et al. 2022). We here document an association between MAs and parasitic infections supporting previous observations (Sayyaf Dezfuli et al. 2021b). Another type of immune cell documented in the current investigation is the mast cell (MC). These cells have a secretory function and are essential components of host immune systems (Reite and Evensen 2006; Sayyaf Dezfuli et al. 2021b; 2025), as they release several types of inflammatory mediators and biogenic amines (histamine and serotonin among others) at the site of infection/inflammation (Galindo-Villegas et al. 2016; Sayyaf Dezfuli et al. 2021b; 2024).

## Conclusion

The present study on experimental infections of rainbow trout with *Anisakis* has for the first time described the cellular local encapsulation process of this parasite’s larvae in rainbow trout. Different host cells involved were shown to be macrophages, neutrophils, mast cells, epithelioid cells, and lymphocytes, but despite this seemingly clear immune response, our gene expression analyses showed a relatively low immune regulation in the central immune organ, the spleen. The study shows that *A. simplex* elicits a cellular immune response, but it is local. Based on evidence from other host-parasite systems, it may be hypothesized that the production of immune-suppressing worm molecules released by the worm larva is suggested to cause downregulation of central immune genes responsible for worm expulsion. This calls for future efforts in exploration of interactions between fish hosts and *A. simplex* larvae, including the possible involvement of excretory/secretory proteins and extracellular particles released by the parasites*.*

## Supplementary Information

Below is the link to the electronic supplementary material.

ESM 1 Microphotograph showing genus characteristics of Anisakis simplex (anterior and caudal parts). Note in frontal part location of excretory pore anterior to nerve ring, ventricle without appendages and absence of intestinal caecum and mucron in caudal end. (PDF 1.15 MB)

ESM 2 Primers and probes used for qPCR assays. All nucleotides are from 5’ end (labeled with FAM) to 3’ end (labeled with BHQ1). All the qPCR assays were optimized to have annealing temperature of 60 °C and having efficiencies of 100% ± 5%. R: indicates reference genes (housekeepers). MS: indicates that the qPCR assay targets both membrane bound and secreted forms. 1: The α chain of IL-12 is common to the two isoforms of IL-12. (PDF 271 KB)

ESM 3 Gene expression results (qPCR) focusing on regulation of immune genes in the spleen of rainbow trout. Comparisons between fold changes in different groups (infected with nematodes and non-infected controls). (PDF 45.0 KB)

## Data Availability

All data are freely accessible.
