# Supplementary figures and images for: Cellular responses in rainbow trout Oncorhynchus mykiss to experimental Anisakis simplex infection
Source: Parasitol Res. 2025 Sep 25;124(9):109. doi: 10.1007/s00436-025-08565-2 (PMC12464046; doi:10.1007/s00436-025-08565-2)

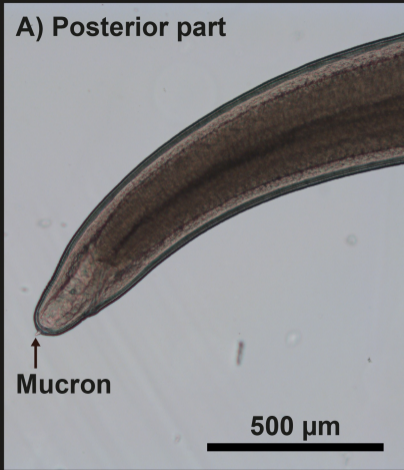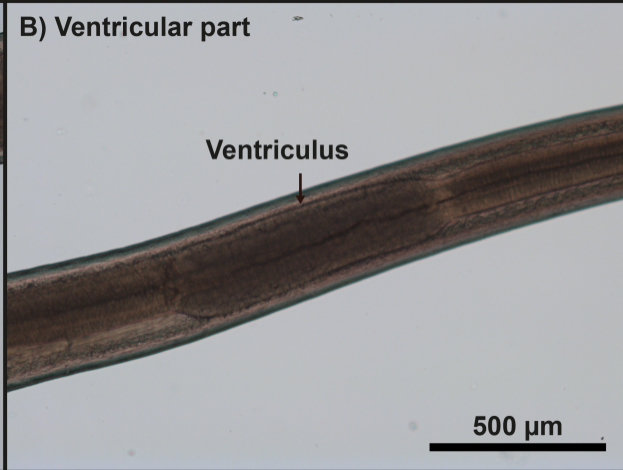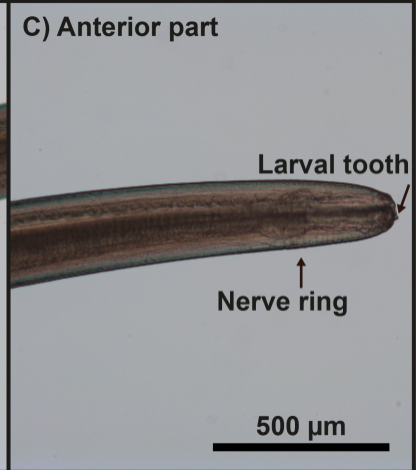

Supplement: Supplementary file 1 — Microphotograph showing genus characteristics of Anisakis simplex (anterior and caudal parts). Note in frontal part location of excretory pore anterior to nerve ring, ventricle without appendages and absence of intestinal caecum and mucron in caudal end. (PDF 1.15 MB) [file 436_2025_8565_MOESM1_ESM.pdf]
