# Supplementary material for: Cellular responses in rainbow trout Oncorhynchus mykiss to experimental Anisakis simplex infection
Source: Parasitol Res. 2025 Sep 25;124(9):109. doi: 10.1007/s00436-025-08565-2 (PMC12464046; doi:10.1007/s00436-025-08565-2)
Supplement: Supplementary file 2 — Primers and probes used for qPCR assays. All nucleotides are from 5’ end (labeled with FAM) to 3’ end (labeled with BHQ1). All the qPCR assays were optimized to have annealing temperature of 60 °C and having efficiencies of 100% ± 5%. R: indicates reference genes (housekeepers). MS: indicates that the qPCR assay targets both membrane bound and secreted forms. 1: The α chain of IL-12 is common to the two isoforms of IL-12. (PDF 271 KB) [file 436_2025_8565_MOESM2_ESM.pdf]

Supplementary File S2 (Table)

Primers and probes used for qPCR assays. All nucleotides are from 5' end (labeled with FAM) to 3' end (labeled with BHQ1). All the qPCR assays were optimized to have annealing temperature of 60°C and having efficiencies of 100% ± 5%. ^{R} indicates reference genes (housekeepers). ^{MS} indicates the qPCR assay targets both membrane bound and secreted forms. ^{1} The α chain of IL-12 is common to the two isoforms of IL-12.

Gene<br>GenBank acc.no.	Length<br>Bp	Primers<br>5'end to 3'end	Probes<br>5'end to 3'end	References
^{R} *ARP*<br>AY505012	106	Fwd: GAAAATCATCCAATTGCTGGATG<br>Rev: CTTCCCACGCAAGGACAGA	CTATCCCAAATGTTTCATTGTCGGCGC	[1]
^{R} *β-actin*<br>AB196465	241	Fwd: ACATCAAGGAGAAGCTGTGCTAC<br>Rev: TACGGATGTCCACGTCACAC	CCTCTCTGGAGAAGAGCTACGAGCTG	[2]<br>Probe [3]
^{R} *ELF-1α*<br>AF498320	63	Fwd: ACCCTCCTCTTGGTCGTTTC<br>Rev: TGATGACACCAACAGCAACA	GCTGTGCGTGACATGAGGCA	[4]
C3.3 & C3.4<br>AF271080 / U61753	85	Fwd: ATTGGCCTGTCCAAAACACA<br>Rev: AGCTTCAGATCAAGGAAGAAGTTC	TGGAATCTGTGTGTCTGAACCCC	[5]
Cathelicidin 1A<br>AY382478	189	Fwd: TCTCTCGTCCTGGGGTT<br>Rev: GTTGTAGCGTGCTGATCTATG	TAATTGGTCGTCCTGGGGGTGG	[3]
Cathelicidin 2A<br>AY360356	135	Fwd: AAAGATTCCAAGGGGGGT<br>Rev: CAAAGGGTGTGTTGTGCTGT	GCTCTCGTCCTGGGTTTGGCTCC	[6]
IFN γ1 and IFN γ2<br>FJ184374 / FJ184375	68	Fwd: AAGGGCTGTGATGTGTTTCTG<br>Rev: TGTACTGAGCGGCATTACTCC	TTGATGGGCTGGATGACTTTAGGA	[7]
IgDm<br>AY870262	304	Fwd: CAGGAGGAAAGTTCGGCATCA<br>Rev: CCTCAAGGAGCTCTGGTTTGGA	CCACACCACACAGACTCTGGCCCTGAA	[8]
IgDs<br>JQ003979	304	Fwd: TGGCACGCCAGGATTTGAC<br>Rev: TCAGAATTGAGTGAACGGACAGACA	CCACACCACACAGACTCTGGCCCTGAA	[8]
^{MS} *IgM*<br>S63348 / AH014877	72	Fwd: ACCCTCCTCTTGGTCGTTTC<br>Rev: TGATGACACCAACAGCAACA	TGATGACACCAACAGCAACA	[7]
^{MS} *IgT*<br>AY870265 / AY870263	73	Fwd: AGCACCAGGGTGAAACCA<br>Rev: GCGGTGGGTTCAGAGTCA	AGCAAGACGACCTCCAAAACAGAAC	[7]
IL-1β<br>AJ223954	91	Fwd: ACATTGCCAACCTCATCATCG<br>Rev: TTGAGCAGGTCCTTGTCCTTG	CATGGAGAGGTTAAAGGGTGGC	[7]
IL-2a<br>FJ571513	110	Fwd: ATGCAACACCACATCAGCAT<br>Rev: TGCCACGGCCCTACAAAAGA	TGCCACGGCCCTACAAAAGA	[3]
IL-4/13a<br>AB574337	138	Fwd: ATCCTTCTCCTCTCTGTTGC<br>Rev: GAGTGTGTGTGTATTGTCCTG	CGCACCGGCAGCATAGAAGT	[9]
IL-6a<br>DQ866150	91	Fwd: ACTCCCCTCTGTCACACACC<br>Rev: GGCAGACAGGTCCTCCACTA	CCACTGTGCTGATAGGGCTGG	[10]
IL-8 isoforms a, b , c, d & e<br>AY160982 to AY160986	69	Fwd: AGAATGTCAGCCAGCCTTGT<br>Rev: TCTCAGACTCATCCCCTCAGT	TTGTGCTCCTGGCCCTCCTGA	[10]
IL-10a<br>AB118099	70	Fwd: CGACTTTAAATCTCCCATCGAC<br>Rev: GCATTGGACGATCTCTTTCTTC	CATCGGAAACATCTTCCACGAGCT	[7]
^{1} *IL-12* α chain<br>HE798148	84	Fwd: CAACGGAACACCACATTCAG<br>Rev: AGCCTGTAGTGAGGCAGCAT	TGCGTGTCTGAGGAACATCCG	[11]
IL-17A/F2a<br>AJ580842	158	Fwd: TCAAAAGCAACGTGTCGAAG<br>Rev: TCCCTCTGATTCCTCTGTGG	TATGCTGCTGGGCCTGACCA	[11]
IL-17c1<br>CAW30792	138	Fwd: CTGGCGGTACAGCATCGATA<br>Rev: GAGTTATATCCATAATCTTCGTATTCGGC	CGTGATGTCCGTGCCCTTTGACGATG	[9]

IL-17c2<br>CAW30793	134	Fwd: CTGGCGGTACAGCATCGATA<br>Rev: CAGAGTTATATGCATGATGTTGGGC	CGTGGTGTCCAGGCCCTTTAATGATG	[9]
IL-22<br>AM748537	64	Fwd: ATGACCACCACCACAGCATT<br>Rev: ATTCCTTTCCCCTCCTCCAT	CTTTCCGCAAGAAGTTGTCCGAG	[12]
Lysozyme<br>X59491	188	Fwd: GAAACAGCCTGCCCAACT<br>Rev: GTCCAACACCACACGCTT	ATACCCAGGCCACCAACCGCAACAC	[13]
SAA<br>AM422446	79	Fwd: GGGAGATGATTCAGGGTTCCA<br>Rev: TTACGTCCCCAGTGGTTAGC	TCGAGGACACGAGGACTCAGCA	[14]
TCR-β<br>AF329700	73	Fwd: TCACCAGCAGACTGAGAGTCC<br>Rev: AAGCTGACAATGCAGGTGAATC	CCAATGAATGGCACAAACCAGAGAA	[7]
TGF-β1a<br>X99303	75	Fwd: TCTGAATGAGTGGCTGCAAG<br>Rev: GGTTTCCCACAATCACAAGG	CTGGAGAGGAGCAGGGATTCCAAT	[7]
TNF-α1 & TNF-α2<br>AJ277604 / AJ401377	75	Fwd: GGGGACAAACTGTGGACTGA<br>Rev: GAAGTTCTTGCCCTGCTCTG	GACCAATCGACTGACCGACGTGGA	[10]

[1] M.K. Purcell, G. Kurath, K.A. Garver, R.P. Herwig, J.R. Winton, Quantitative expression profiling of immune response genes in rainbow trout following infectious haematopoietic necrosis virus (IHNV) infection or DNA vaccination, Fish & shellfish immunology 17(5) (2004) 447-462.
[2] S.H. Sugiura, K. Kelsey, R.P. Ferraris, Molecular and conventional responses of large rainbow trout to dietary phosphorus restriction, Journal of comparative physiology. B, Biochemical, systemic, and environmental physiology 177(4) (2007) 461-72.
[3] S. Zuo, A.M. Karami, J. Ødegård, H. Mathiessen, M.H. Marana, R.M. Jaafar, L. von Gersdorff Jørgensen, M. Abdu, P.W. Kania, I. Dalsgaard, T. Nielsen, K. Buchmann, Immune gene expression and genome-wide association analysis in rainbow trout with different resistance to Yersinia ruckeri infection, Fish & shellfish immunology 106 (2020) 441-450.
[4] H.-C. Ingerslev, E.F. Pettersen, R.A. Jakobsen, C.B. Petersen, H.I. Wergeland, Expression profiling and validation of reference gene candidates in immune relevant tissues and cells from Atlantic salmon (Salmo salar L.), Molecular Immunology 43(8) (2006) 1194-1201.
[5] M.K. Raida, K. Buchmann, Innate immune response in rainbow trout (*Oncorhynchus mykiss*) against primary and secondary infections with *Yersinia ruckeri* O1, Developmental and comparative immunology 33(1) (2009) 35-45.
[6] J. Xueqin, P.W. Kania, K. Buchmann, Comparative effects of four feed types on white spot disease susceptibility and skin immune parameters in rainbow trout, *Oncorhynchus mykiss* (Walbaum), Journal of Fish Diseases 35(2) (2012) 127-135.
[7] M.K. Raida, K. Buchmann, Temperature-dependent expression of immune-relevant genes in rainbow trout following *Yersinia ruckeri* vaccination, Diseases of Aquatic Organisms 77(1) (2007) 41-52.
[8] J. Skov, J.K. Chettri, R.M. Jaafar, P.W. Kania, I. Dalsgaard, K. Buchmann, Effects of soluble immunostimulants on mucosal immune responses in rainbow trout immersion-vaccinated against Yersinia ruckeri, Aquaculture 492 (2018) 237-246.
[9] J.K. Chettri, J.A. Kuhn, R.M. Jaafar, P.W. Kania, O.S. Moller, K. Buchmann, Epidermal response of rainbow trout to Ichthyobodo necator: immunohistochemical and gene expression studies indicate a Th1-/Th2-like switch, J Fish Dis 37(9) (2014) 771-83.
[10] M.K. Raida, K. Buchmann, Bath vaccination of rainbow trout (*Oncorhynchus mykiss* Walbaum) against *Yersinia ruckeri*: effects of temperature on protection and gene expression, Vaccine 26(8) (2008) 1050-62.
[11] R.M. Jaafar, J.K. Chettri, I. Dalsgaard, A. Al-Jubury, P.W. Kania, J. Skov, K. Buchmann, Effects of adjuvant Montanide™ ISA 763 A VG in rainbow trout injection vaccinated against Yersinia ruckeri, Fish & Shellfish Immunology 47(2) (2015) 797-806.

[12] M.M. Olsen, P.W. Kania, R.D. Heinecke, K. Skjoedt, K.J. Rasmussen, K. Buchmann, Cellular and humoral factors involved in the response of rainbow trout gills to *Ichthyophthirius multifiliis* infections: Molecular and immunohistochemical studies, Fish & Shellfish Immunology 30(3) (2011) 859-869.
[13] J.K. Chettri, M.K. Raida, P.W. Kania, K. Buchmann, Differential immune response of rainbow trout (Oncorhynchus mykiss) at early developmental stages (larvae and fry) against the bacterial pathogen Yersinia ruckeri, Developmental & Comparative Immunology 36(2) (2012) 463-474.
[14] J. Skov, P.W. Kania, L. Holten-Andersen, B. Fouz, K. Buchmann, Immunomodulatory effects of dietary beta-1,3-glucan from Euglena gracilis in rainbow trout (Oncorhynchus mykiss) immersion vaccinated against Yersinia ruckeri, Fish & shellfish immunology 33(1) (2012) 111-20.
