# Supplementary material for: Cellular responses in rainbow trout Oncorhynchus mykiss to experimental Anisakis simplex infection
Source: Parasitol Res. 2025 Sep 25;124(9):109. doi: 10.1007/s00436-025-08565-2 (PMC12464046; doi:10.1007/s00436-025-08565-2)
Supplement: Supplementary file 3 — Gene expression results (qPCR) focusing on regulation of immune genes in the spleen of rainbow trout. Comparisons between fold changes in different groups (infected with nematodes and non-infected controls). (PDF 45.0 KB) [file 436_2025_8565_MOESM3_ESM.pdf]

	N	Student's t-test	Fold
Thresholds	3	0,05	±2

Gene	Unifected Control			Student's	Anisakis infected		
	N	Fold	GSE	t-test	N	Fold	GSEM
C3	6	1	1,15	0,1307	6	-1,73	1,48
Cathelicidin 1	9	1	1,53	0,3839	6	-1,28	1,97
Cathelicidin 2	10	1	1,77	0,4099	7	1,21	1,55
IFNγ	10	1	1,19	0,4923	8	-1,01	1,24
IgDm	10	1	1,14	0,0268	8	-1,63	1,21
IgDs	10	1	1,17	0,0003	8	-2,84	1,19
IgM	10	1	1,17	0,0169	8	-1,95	1,26
IgT	7	1	1,33	0,4398	8	-1,06	1,22
IL-1ß	10	1	1,40	0,2866	8	1,42	1,62
IL-2	10	1	1,15	0,0489	8	-1,99	1,45
IL-4/13a	10	1	1,16	0,1067	8	-1,47	1,29
IL-6	10	1	1,21	0,1503	8	1,58	1,46
IL-8	10	1	1,13	0,0365	8	1,88	1,37
IL-10	10	1	1,20	0,0178	8	2,44	1,41
IL-22	3	1	1,13	0,1136	5	-1,89	1,36
IL-17C2	5	1	1,19	0,1741	6	-1,27	1,15
Lysozyme	10	1	1,13	0,0024	8	-3,61	1,47
SAA	10	1	1,42	0,4982	7	1,00	1,57
TGF ß	10	1	1,11	0,0326	8	-1,42	1,14
TNF α	10	1	1,20	0,2919	8	1,34	1,66
IL-12	0	NA	NA	0,4444	1	NA	NA
IL-17AF2	0	NA	NA	0,5000	0	NA	NA
IL-17C1	0	NA	NA	0,1830	2	NA	NA
TCR ß	0	NA	NA	0,1830	2	NA	NA

←Mann-Whitney test (IL-12)
←Mann-Whitney test (IL-17AF2)
←Mann-Whitney test (IL-17C1)
←Mann-Whitney test (TCR ß)

Folds are calculated as geometrical means

GSEM indicates geometrical standard error of mean

NA indicates Not Available

In case of 4 genes, less than 3 Cq values were obtained in at least one of the groups tested. Then a qualitative approach was used by using presence / absence of Cq values in the non-parametric Mann-Whitney test
